# Highly Selective, ppb-Level Xylene Gas Detection by Sn^2+^-Doped NiO Flower-Like Microspheres Prepared by a One-Step Hydrothermal Method

**DOI:** 10.3390/s19132958

**Published:** 2019-07-04

**Authors:** Shaohe Lu, Xuefeng Hu, Hua Zheng, Junwen Qiu, Renbing Tian, Wenjing Quan, Xinjie Min, Peng Ji, Yewei Hu, Suishi Cheng, Wei Du, Xiaoqiang Chen, Beiliang Cui, Xiaorong Wang, Wei Zhang

**Affiliations:** 1 State Key Laboratory of Materials-Oriented Chemical Engineering, College of Chemical Engineering, Nanjing Tech University, No. 5 XinMofan Road, Nanjing 210009, China; 2 School of Instrument Science and Opto-Electronics Engineering and Research Center for Sensor Science and Technology, Hefei University of Technology, No. 193 Tunxi Road, Hefei 230009, China; 3 School of Electrical Engineering & Intelligentization, Dongguan University of Technology, No. 1 Daxue Rd, Dongguan 523808, China; 4 Network Information Center, Nanjing Tech University, No. 5 XinMofan Road, Nanjing 210009, China; 5 College of Electrical Engineering and Control Science, Nanjing Tech University, No. 5 XinMofan Road, Nanjing 210009, China

**Keywords:** one-step hydrothermal, Sn^2+^-doped NiO, flower-like microsphere, gas sensor, xylene

## Abstract

Detecting xylene gas is an important means of avoiding human harm from gas poisoning. A precise measurement demands that the gas sensor used must have high sensitivity, high selectivity, and low working temperature. To meet these requirements, in this study, Sn^2+^-doped NiO flower-like microspheres (SNM) with different amounts of Sn^2+^ synthesized by a one-step hydrothermal process were investigated. The responses of gas sensors based on different Sn^2+^-doped NiO materials for various targeting gases were fully characterized. It was found that all of the synthesized materials exhibited the best gas response at a working temperature of 180 degrees, which was much lower than the previously reported working temperature range of 300–500 degrees. When exposed to 10 ppm xylene, the 8 at% Sn^2+^-doped NiO sensor (mol ratio) exhibited the highest response, with a value of 30 (R_g_/R_a_). More significantly, the detection limit of the 8 at% Sn^2+^-doped NiO sensor for xylene is down in the ppb level. The Sn^2+^-doped NiO material also exhibits excellent selectivity for other gases with long-term stability and repeatability. The significant improvement in the response to xylene can theoretically be attributed to a decrease in the intrinsic hole carrier concentration, higher amounts of adsorbed oxygen and active sites.

## 1. Introduction

Xylene is an important solvent widely used in the ink, paint, adhesives, and pigment industries [1]. Although the damage caused by exposure to low concentrations of xylene (<1 ppm) might be reversible, prolonged exposure can lead to headaches, irritation of the skin, short-term hearing and memory loss [2]. Furthermore, with exposure to 100 ppm, one may immediately feel dizzy. An exposure to more than 200 ppm, can lead to vomiting [3,4]. Therefore, to ensure a healthy working environment, the accurate in situ detection of xylene is essential.

Semiconductor metal oxides, such as ZnO [5], SnO_2_ [6], WO_3_ [7], and NiO [8], have the advantages of high sensitivity, fast response, and easy integration with signal processing circuits, and they have been extensively studied for the detection of harmful and toxic gases [9]. In general, compared to n-type oxide semiconductors, which exhibit high responses to ethanol and formaldehyde instead of xylene and toluene, p-type semiconductor oxides react preferable with xylene and toluene due to their superior ability to oxidize methylbenzenes [10]. Specifically, nickel oxide (NiO) is a wide energy gap (3.6 to 4.0 eV) p-type semiconductor with good chemical and thermal stability [11,12]. Currently, the major challenges for gas sensors based on semiconductor metal oxides are the selectivity of the gas detection and the stability during high-temperature operation. Doping NiO with other metal oxides can improve the operating temperature and raise the sensitivity of the gas response [13,14]. However, limited by the working principle of the semiconductor gas sensor, it is difficulty to solve the existing selectivity issues by metal oxide doping only.

The doping of supported noble metal nanoclusters or nanoparticles, such as Pd, Pt, Au, Rh and Ag nanoparticles, into some pure metal oxides can enhance their catalytic performance by changing the chemical interactions between the analyte and metal oxide, improving the catalysis of the gas-sensitive material, manipulating the adsorption-desorption energy, and decreasing the response and recovery times [2,7]. Considering the high price of precious metals, nonprecious metal doping is economically attractive, but the key is how to achieve the high selectivity and sensitivity requirements. Kim et al. [15] successfully synthesized Sn^4+^-doped NiO microspheres that exhibited a high response and selectivity for xylene compared to pure NiO at an optimal temperature of 300 °C. Feng et al. [16] demonstrated that the response of NiO nanotubes under 50 ppm xylene exposure at 325 °C was enhanced by nearly 63 times via Cr^3+^ doping. Gao et al. [17] also provided evidence that the sensing performance of NiO microspheres for xylene at an optimal temperature of 250 °C can be enhanced by Sn^2+^ doping. Although improved material syntheses and metal atom doping have indeed improved the detection limit and reduced the operating temperature of xylene sensors, the selectivity of the measurement remains a challenge. Another striking problem is that the current methods of material synthesis involve multiple steps. These complex multistep synthesis methods might introduce more parameters that could alter the metal ion environment, making it more difficult to control the ion ratios, bonding, defects, and surface structures. In practical applications, the first requirement for industrialization is to determine the most simplified method of synthesizing a material. Therefore, further investigations to improve the detection sensitivity and selectivity, lower the working temperature and synthesize NiO materials doped with different elements via simple methods are extremely desirable. 

In this work, a series of different Sn^2+^-doped NiO microspheres (SNM) were synthesized by a one-step facile hydrothermal method. The gas sensor performance indicates that at an optimal molar ratio of Sn^2+^/Ni^2+^ (8%), the response of SNM sensor to 10 ppm xylene is enhanced 21 times more than that of the pure NiO sensor. The lower limit of measurement is on the order of ppb. Meanwhile, the optimal working temperature (T_w_) of the 8 at% SNM is 180 °C. In addition, the extremely high selectivity for other solvent gases (toluene, acetone and ethanol) and no solvent gases (formaldehyde, ammonia and hydrogen) were demonstrated. Furthermore, the mechanisms for the enhanced performance were also discussed. This research is expected to open up new avenues of research for detecting solvent gases with high sensitivity and selectivity at low temperatures.

## 2. Material Preparing Methods

### 2.1. Synthesis of the Sn^2+^-Doped NiO Microspheres

All chemicals in this work were high analytical grade and used without further purification. The pure NiO and SNM were synthesized by a one-step hydrothermal method. To synthesize the SNM, Ni(CH_3_COO)_2_·4H_2_O (0.186 g) and a given amount of SnCl_2_·2H_2_O (the molar ratios of Sn^2+^/Ni^2+^ were 2.0, 4.0, 8.0 and 12.0 at%) were poured into methanol (20 mL) under constant stirring for 20 min at room temperature (RT). Then, a methanol solution (5 mL) containing polyvinylpyrrolidone (PVP, 0.02 g) was added dropwise to the mixture, which was then transferred to 50 mL Teflon-lined stainless steel autoclave, stirred for 15 min and heated at an electric oven up to 180 °C for 24 h. After the autoclave had naturally cooled to RT, the reactants were collected, washed, and then dried at 60 °C in air for 24 h. Finally, the SNM were annealed at 500 °C for 2 h in air. The pure NiO microspheres were prepared using a similar method to that used for the SNM, except no SnCl_2_·2H_2_O was added. In the following discussion, the SNM with different molar ratios of Sn^2+^/Ni^2+^ are denoted as SNM-x, where x refers to the molar ratio of Sn^2+^/Ni^2+^.

### 2.2. Structural Characterization

The crystal structure of the SNM were characterized by powder X-ray diffraction (XRD) using a monochromatized Cu target radiation source (standard Cu Kα radiation, λ = 1.5418 Å, D8 Advance, Bruker Corporation, Brucker, Germany). XRD scan range is from 30 to 80 degrees. The morphological and elemental mapping of the SNM were performed by field emission scanning electron microscopy (SEM) (FE-SEM, Quanta 200, FEI, Tokio, Japan). The nano-scale microstructures were characterized by high-resolution transmission electron microscopy (HRTEM) at a working voltage of 200 kV (JEM-2100F, Tokio, Japan). The surface chemical composition and electronic valence state of the products were determined by X-ray photoelectron spectroscopy (XPS, ESCALAB 250Xi, Thermo, Waltham, MA, USA) with a monochromatic Al Kα X-ray source (1486.6 eV). The textural properties of the as-deposited powders, such as the specific surface area, were studied by the Brunauer-Emmett-Teller (BET) method (Quanta Autosorb-3B).

### 2.3. Fabrication and Measurements of the Sensors

**Figure 1:**
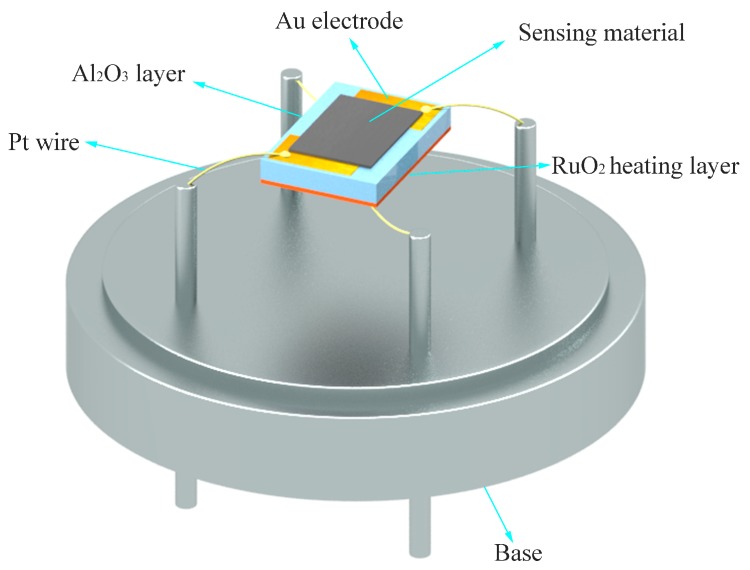
Schematic diagram of the packaged gas sensor.

The as-prepared SNM powders were added into ethanol solution. After three minutes stirring, SNM were evenly distributed, forming a paste, and then drop-coated on an alumina substrate with two L gold electrodes on its top surface and a ruthenium oxide heating layer on its bottom surface. A schematic of the sensor is shown in Figure 1.

To remove residual ethanol and improve sensor’s stability and reliability, the sensors were aged at 240 °C for 7 days in air. The response of gas sensors to exposure various gases was performed by an integrated test platform (LW-GS-002, Six-dimension Sensor Technology, Ltd., Nanjing, China). The testing apparatus is equipped with two mass flow meters (MFs) to control the gas concentration and highly purified air served as the diluent and carrier gas. The sensor’s response to reducing gases was defined as the ratio (S = R_g_/R_a_) of the resistance of the sensor in a given concentration of the target gas (R_g_) to that in air (R_a_). The response time in this experiment is defined as the time for the sensor to rise 90% of the R_g_. In our work, we normally take three samples for our performance characterizations, the first one for screening to find the best sample, the second one for confirmation and the third one for a repeatability test.

## 3. Results

### 3.1. Morphological and Structural Analyses

**Figure 2:**
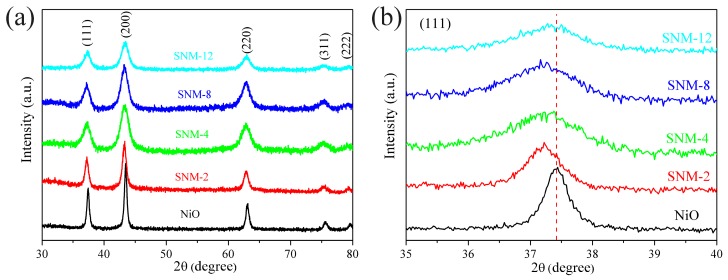
XRD patterns for the pure NiO and the 2, 4, 8, and 12 at% SNM, (**a**) full scale scan from 30–80 degree and (**b**) expanding pattern at (111) peak for these materials.

Full XRD patterns of the pure NiO and SNM are exhibited in Figure 2. All the diffraction peaks of the pure NiO and SNM samples can be attributed to a face-centered-cubic NiO phase (Fm-3m, JCPDS No. 47-1049), and no other structural phase is observed. The (111) peaks of the pure NiO and SNM shown in Figure 2b reveal that the peaks of all the SNM shift slightly to lower angles, indicating that the Sn^2+^ doping on the host NiO can slightly expand NiO lattice parameter. These observed results are consistent with literature reports of Sn doped NiO samples (loaded with 1.5 at% Sn^2+^) [17]. These results might be due to the incorporation of Sn^2+^ into the interstitial positions of the NiO lattice because the radius of Sn^2+^ with a coordination number of 6 is 1.18 Å, which is larger than that of Ni^2+^, 0.69 Å, with the same coordination number. Accordingly, the Sn^2+^ dopants prefer to occupying the interstitial positions [17,18]. In addition, the diffraction peaks becoming broader with increasing Sn^2+^ amount indicates a decrease in the crystallite size. According to the Scherrer equation (D = 0.89λ/βcosθ), the average crystal sizes of the pure NiO and the 2, 4, 8, and 12 at% SNM are 18.2, 14.3, 12.6, 10.9, and 10.0 nm, respectively. More Sn^2+^ doping may prevent NiO crystallites from further grain growth.

**Figure 3:**
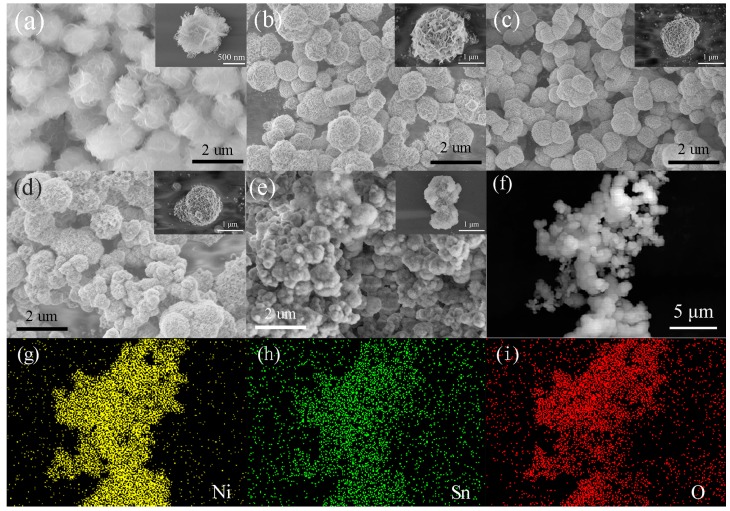
(**a**–**e**) Panoramic SEM images of the NiO, SNM-2, SNM-4, SNM-8, and SNM-12 samples, respectively; (**f**–**i**) SEM image and the elemental mapping of the Ni, Sn and O in the SNM-8 sample.

The shape and composition of the as-prepared pure NiO and SNM were examined by SEM. As shown in Figure 3, the SEM images of the pure NiO samples reveal the presence of flower-like microspheres with uniform distribution and an average diameter of ~1.5 μm. A single flower-like NiO microsphere shown in the inset of Figure 3a reveals that the microsphere consists of multiple curving low-dimensional nanosheets with porous surfaces. The morphologies of SNM-2, SNM-4, SNM-8, and SNM-12 are also shown in Figure 3b–e, respectively. These samples have a slightly lower dispersity and relatively poor uniformity in diameter in comparison with the pure NiO. Furthermore, as the concentration of Sn^2+^ increases, SNM become denser. The corresponding single microspheres for the SNM samples are also shown in the insets of Figure 3b–e. In addition, the elemental mapping of SNM-8 shown in Figure 3f–i reveals that all three main elements, i.e., nickel (Ni), tin (Sn), and oxygen (O), are uniformly distributed, indicating that Sn^2+^ is successfully doped into whole NiO microspheres.

**Figure 4:**
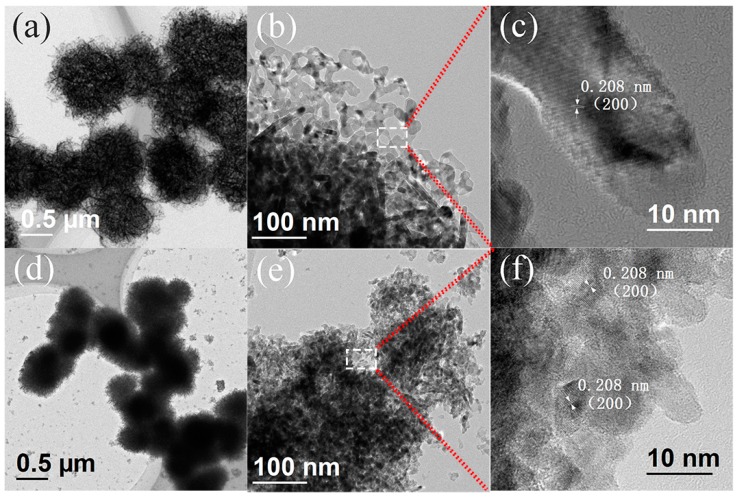
(**a**,**b**) Typical TEM and (**c**) HRTEM images of NiO sample; (**d**,**e**) Typical TEM and (**f**) HRTEM images of the SNM-8 sample.

The TEM and HRTEM measurements of the NiO and SNM-8 samples were performed to further investigate the morphologies and crystallographic features, and the results are shown in Figure 4a–c and Figure 4d–f, respectively. 

Figure 4a,b show that the flower-like NiO microspheres consist of hundreds of nanosheets with random, intertwined porous structures, which is consistent with previous SEM results. The formation of porous microstructure might result from the thermal decomposition of the precursor, which releases a stored gases during the annealing process. Furthermore, as shown in Figure 4c, the HRTEM image of NiO exhibits that the interplanar spacing of 0.208 nm is assigned to the (200) lattice plane of NiO. In addition, Figure 4d,e show that the nanosheets of SNM-8 that form the nanoparticles and microspheres are indeed denser and smaller than those of the pure NiO. Moreover, the HRTEM image shown in Figure 4f indicates that the grain size of SNM-8 is smaller than that of the pure NiO, which is consistent with the observation at XRD.

**Figure 5:**
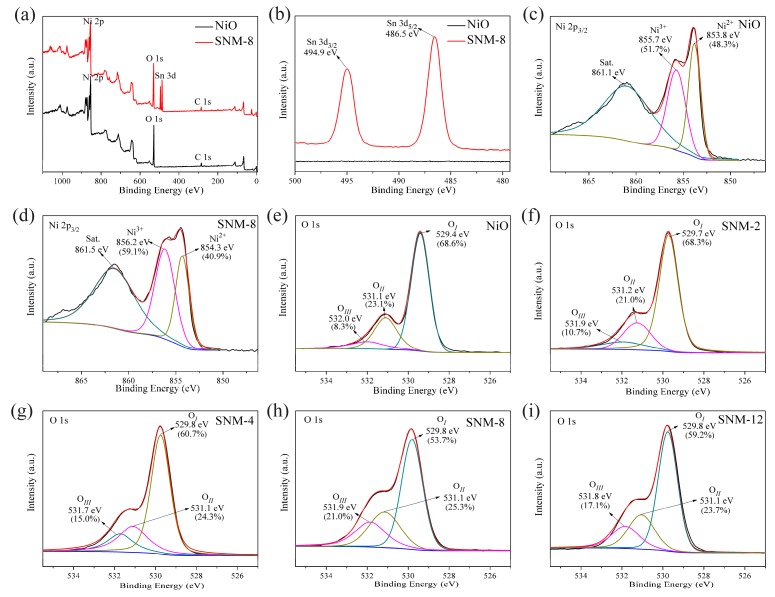
(**a**) Survey XPS patterns of the pure NiO and SNM-8 samples; (**b**) Sn 3d spectrum of the SNM-8 sample; (**c**,**d**) Ni 2p_3/2_ high-resolution XPS spectra of the pure NiO and SNM-8, respectively; (**e**–**i**) O 1s high-resolution XPS spectra of the pure NiO, SNM-2, SNM-4, SNM-8, and SNM-12 samples, respectively.

The composition and physicochemical state of NiO and SNM-8 were analyzed by XPS. As shown in Figure 5, from the survey spectra of the NiO and SNM-8, Ni, Sn, O and C (carbon) can all be clearly observed. The weak C peak is attributed to adventitious carbon adsorbed on the surface of the samples as a contaminant, and the C 1s binding energy of 284.7 eV was used as the reference for calibration. As shown in the Sn 3d spectrum in Figure 5b, the two nearly symmetric peaks located at 494.9 eV and 486.5 eV are attributed to Sn 3d_3/2_ and Sn 3d_5/2_ of Sn^2+^, respectively [17,19], indicating the presence of Sn^2+^ in SNM-8. More accurate scans of the Ni 2p_3/2_ of the pure NiO and SNM-8 are shown in Figure 5c and Figure 5d, respectively. For the pure NiO sample (Figure 5c), the Ni 2p_3/2_ spectrum is deconvoluted into peaks at 853.8, 855.7 and 861.1 eV, corresponding to the binding states of Ni^2+^, Ni^3+^, and its satellite peak, respectively. As shown in Figure 5d, for SNM-8, the binding energies of Ni^2+^ and Ni^3+^ are clearly observed at 854.3 and 856.2 eV, respectively, and they are both shifted by 0.5 eV to higher energies. This shift reflects the change in the interactions between Ni and O due to Sn^2+^ doping. Since the electronegativity of Ni (χ = 1.91) is lower than that of Sn (χ = 1.96) [15], it confirms the doping of Sn^2+^ into NiO. The ratio of Ni^3+^/Ni^2+^ to the pure NiO and SNM-8 are calculated to be 1.07 and 1.44, respectively. With the doping Sn^2+^ into the NiO nanocrystals, the ratio of Ni^3+^/Ni^2+^ increases, indicating that the Ni^2+^ ions are partially oxidized to Ni^3+^ as an affection of Sn^2+^ doping.

It was reported that oxygen adsorbed on NiO can oxidize Ni^2+^ to Ni^3+^, giving negatively charged oxygen [20]. An excess of negatively charged interstitial oxygens (Oi″) can also be attributed to the formation of Ni^3+^ [21]. Thus, the increase in the Ni^3+^/Ni^2+^ ratio affected by Sn^2+^ doping can result from the formation of negatively charged oxygen at an interstitial site from an oxygen molecule as it withdraws electrons from Ni^2+^, as shown in Equation (1). That is, the higher ratio of Ni^3+^/Ni^2+^ for SNM-8 might indicate that it has more negatively charged oxygens than the pure NiO.

(1) $$\frac{1}{2}O_{2}(g)+2\mathrm{NiO}\overset{2\mathrm{NiO}}{\to }O_{i}^{\prime \prime }+2{\mathrm{Ni}}_{\mathrm{Ni}}^{•}+2O_{O}^{\times }$$

To determine the oxygen chemical states and variations in the relative contents of different oxygen species in the gas sensing materials, the high-resolution O 1s XPS spectra were characterized. Figure 5e–i show that the O 1s spectra are fitted to three peaks at 529.4 ± 0.4, 531.1 ± 0.1, and 532.0 ± 0.3 eV corresponding to the oxygen (O_*I*_) lattice, oxygen-deficient regions (O_*II*_), and chemisorbed oxygen species (O_*III*_), respectively. The formation of lattice oxygen is attributed to Ni-O or Ni-O-Sn, because O^2−^ ions are present in the metal oxides. The oxygen-deficient regions can be assigned to O^2−^ ions in the oxygen-deficient regions. The chemisorbed oxygen can be made by chemisorbed or dissociated oxygen species (O^−^) adsorbed on the surface of the material [18]. Additionally, the contents of different oxygen species, i.e., the relative percentage weight of the three peaks (O_*I*_, O_*II*_, and O_*III*_) for the pure NiO are 68.6, 23.1 and 8.3%, respectively, where for the SNM-8, the percentage are 53.7, 25.3 and 21.0%, respectively. Therefore, the contents of the O_*II*_ and O_*III*_ portion increase after Sn^2+^ doping. In particular, the O_*III*_ component (21.0%) of the SNM-8 is ~2.5 times higher than that of the pure NiO (8.3%). Interestingly, compared to the pure NiO, the sums of O_*II*_ and O_*III*_ for all the SNM are higher than that of the pure NiO. The relative contents gradually increase with improving the ratio of Sn^2+^/Ni^2+^, which reach the peak values (25.3% and 21.0%, respectively) at the ratio of 8 at%. This result is consistent with the fact that the SNM-8 sensor exhibits a better gas response than that of the pure NiO and other doping materials. Generally, the number of chemisorbed oxygen species that can participate in redox reactions at sensing process increases as the content of the O_*III*_ ions increases. Meanwhile, the interstitial oxygen and oxygen vacancies contributing to the O_*II*_ component can provide active sites for the sensing materials to facilitate gas adsorption and reaction [15,22,23]. Therefore, based on the O 1s XPS results, the enhanced sensing properties of the SNM-8 might result from the increase in the chemisorbed oxygen species as a result of Sn^2+^ doping.

**Figure 6:**
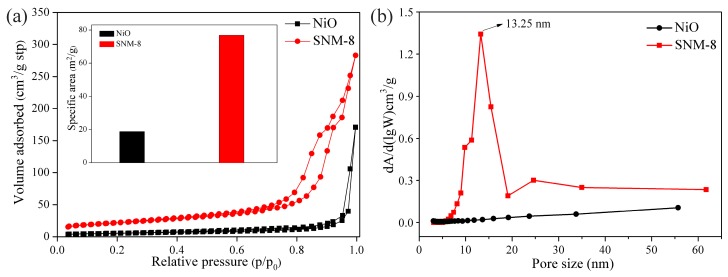
(**a**) N_2_ adsorption-desorption isotherms of the NiO and SNM-8 samples, and the inset is the corresponding BET specific surface areas of the NiO and SNM-8 samples; (**b**) Pore size distribution curves of the NiO and SNM-8 samples.

Since the porous structure at the surface of a sensing material greatly influences the gas sensing properties, the pore size distribution and specific surface area of the NiO and SNM-8 were measured from their N_2_ adsorption-desorption isotherms. As shown in Figure 6a, the N_2_ adsorption- desorption curves of the NiO and SNM-8 are typical type V isotherms with H_3_ hysteresis loops [24]. The BET specific surface area of the SNM-8 (76.9 m^2^/g) is four times higher than that of the pure NiO (18.9 m^2^/g), indicating that Sn^+2^ doping into NiO can result in an increase in the specific surface area. The amounts of adsorbed gas increases with the specific surface area due to more adsorption sites created, which is consistent with the XPS results. In addition, Figure 6b shows that the majority of the pores of the SNM-8 are less than 20 nm in size, and the peak at approximately 13 nm in the pore size distribution curve might correspond to the gap between the nanosheets of SNM-8. Regarding the hysteresis loop of the pure NiO in the high-pressure range (0.8 < P/P_0_ < 1), the powders might contain disordered mesopores and macropores according to the IUPAC classification [25,26]. Therefore, the pore size distribution of the pure NiO is disordered. In addition, the total pore volume of the SNM-8 (0.445 cm^3^/g) is larger than that of the pure NiO (0.264 cm^3^/g), which means that the SNM-8 is more accessible to gases.

### 3.2. Gas Sensing Properties

**Figure 7:**
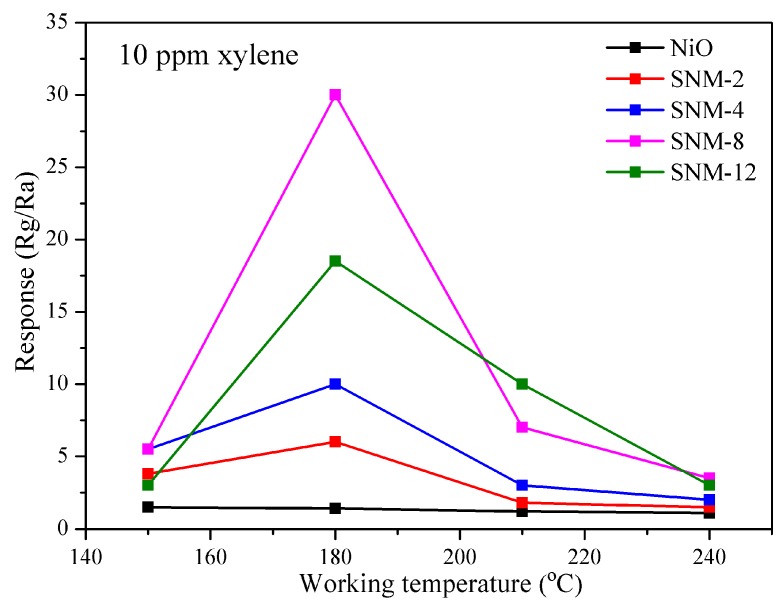
Gas responses of the NiO and SNM sensors toward 10 ppm xylene at different working temperatures.

To demonstrate how doping Sn^2+^ into the pure NiO affects the gas sensing properties, the sensors based on the pure NiO and SNM materials were characterized in detail. Because both the T_w_ and doping amount greatly influence the sensing behavior, the gas sensing responses of the pure NiO and SNM with different Sn^2+^ doping amounts as a function of T_w_ were first explored. Figure 7 shows the xylene gas response at a concentration of 10 ppm at the T_w_ range of 150 to 240 °C for various amounts of Sn^2+^ doping. The responses of all the SNM exhibit a “volcano” shape, and the optimal T_w_ of each SNM sensor is 180 °C. Of all the SNM, SNM-8 has the overall highest response over the entire T_w_ range. The unusual effect of T_w_ on the gas response can be explained by the competing thermal effects on molecular adsorption and desorption. At a lower T_w_, the gas molecules have a low chemical activation, leading to an inert response. As T_w_ increases, the thermal energy of the gas molecules increases, which can help them overcome the activation energy barrier of the reaction. On the other hand, if T_w_ is further increased, the adsorbed gas molecules might escape before reacting with the active centers at the surface, resulting in a poor response [27].

**Figure 8:**
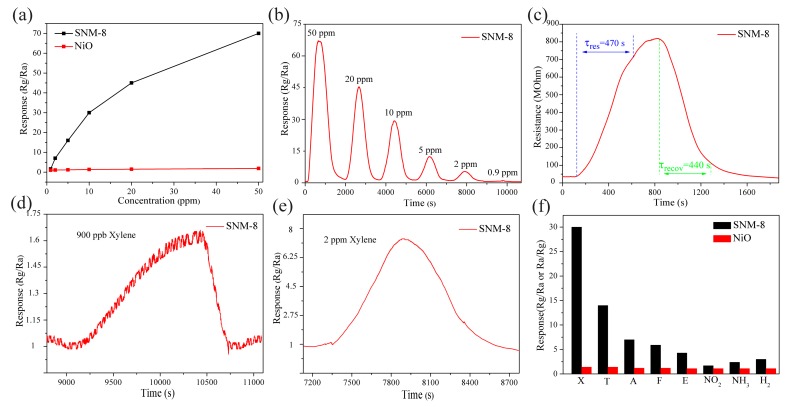
(**a**) Responses of the NiO and SNM-8 sensors as a function of the xylene concentration at 180 °C; (**b**) Dynamic response of the SNM-8 sensor to 0.9-50 ppm xylene at 180 °C; (**c**) Response and recovery times of the SNM-8 sensor to 10 ppm xylene at 180 °C; (**d**,**e**) Response curve of the SNM-8 sensor to 900 ppb and 2 ppm xylene at 180 °C, respectively; (**f**) Gas responses of the SNM-8 sensor to 10 ppm of various VOCs (X, xylene; T, toluene; A, acetone; F, formaldehyde; E, ethanol), 10 ppm NO_2_, 100 ppm NH_3_, and 100 ppm H_2_ at 180 °C.

The sensor response of the SNM-8 versus the xylene concentration is shown in Figure 8a. The response of the SNM-8 increases sharply as the concentration of xylene increases from 900 ppb to 20 ppm, and the growth rate gradually slows down above 20 ppm. In contrast, the response of the pure NiO-based sensor hardly changes with the xylene concentration. These results demonstrate that the performance of the SNM-8-based sensor is much better than that of the pure NiO sensor. As shown in detail in Figure 8b, the SNM-8 sensor exhibits responses (R_g_/R_a_) of 70 and 30 to 50 ppm and 10 ppm xylene, respectively, at 180 °C. Both of these responses are nearly 20 times higher than that of the pure NiO at the same xylene concentrations. Figure 8c shows that the responding and recovering times of the SNM-8 sensor toward 10 ppm xylene at 180 °C are 470 and 440 s, respectively. As shown in Figure 8d,e, upon exposure to the low xylene concentrations of 900 ppb and 2 ppm, the responses of the SNM-8 sensor are approximately 1.6 and 7, respectively. To our knowledge and based on a comparison with the currently reported values listed in Table 1, this work may be the first to report the detection of a ppb-level xylene concentration at the low T_w_ of 180 °C.

Selectivity is also an important evaluation parameter of gas sensors performance. Figure 8f shows the gas responses of the NiO and SNM-8 sensors to various solvent gases (X-xylene, T-toluene, A-acetone, E-ethanol) and non-solvent gases (F-formaldehyde, NO_2_, NH_3_, and H_2_) at the T_w_ of 180 °C. A very low response to all the target gases can be seen for pure NiO sensor thus it has poor selectivity. In contrast, the overall enhanced responses of the SNM-8 sensor to all the target gases are addressed, i.e., the response to 10 ppm xylene is 30, whereas the responses to 10 ppm toluene, 10 ppm acetone, 10 ppm formaldehyde, 10 ppm ethanol, 10 ppm NO_2_, 100 ppm NH_3_, and 100 ppm H_2_ are 14, 7, 5.9, 4.3, 1.7, 2.4, and 3, respectively. Therefore, the SNM-8 sensor exhibits a high selectivity to xylene. Generally, the selectivity of a sensor is influenced by many factors such as the morphology of the materials, the lowest unoccupied molecule orbit energy of gas molecules, the amount of gas adsorption on the sensing materials, and the T_w_. As for the SNM-8 sensor, the optimal doping of Sn^2+^ may extend the difference of adsorption energy for different gases and magnify the effect from T_w_, which means the best response to xylene for the SNM-8 sensor is at the T_w_ of 180 °C while the best response to other gases may be at different temperatures.

**Figure 9:**
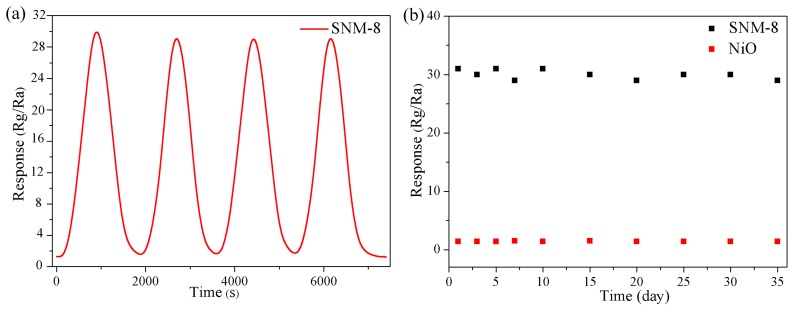
(**a**) Reproducibility of the response of SNM-8 to 10 ppm xylene at 180 °C; (**b**) Long-term stability tests of the NiO and SNM-8 sensors to 10 ppm xylene at 180 °C.

Another important aspect of gas sensors for practical applications is the repeatability and long-term stability of the sensor material. The reproducibility and long-term stability of the SNM-8 sensor in detecting 10 ppm xylene at 180 °C were examined, as shown in Figure 9. Figure 9a shows that the response of the SNM-8 sensor remains stable after four cycles. Figure 9b shows that during the 30-day test, the responses only vary slightly near the average value of 30, indicating the good stability of the SNM-8 sensor.

### 3.3. Mechanism of the Enhanced Gas Sensing Performance

**Figure 10:**
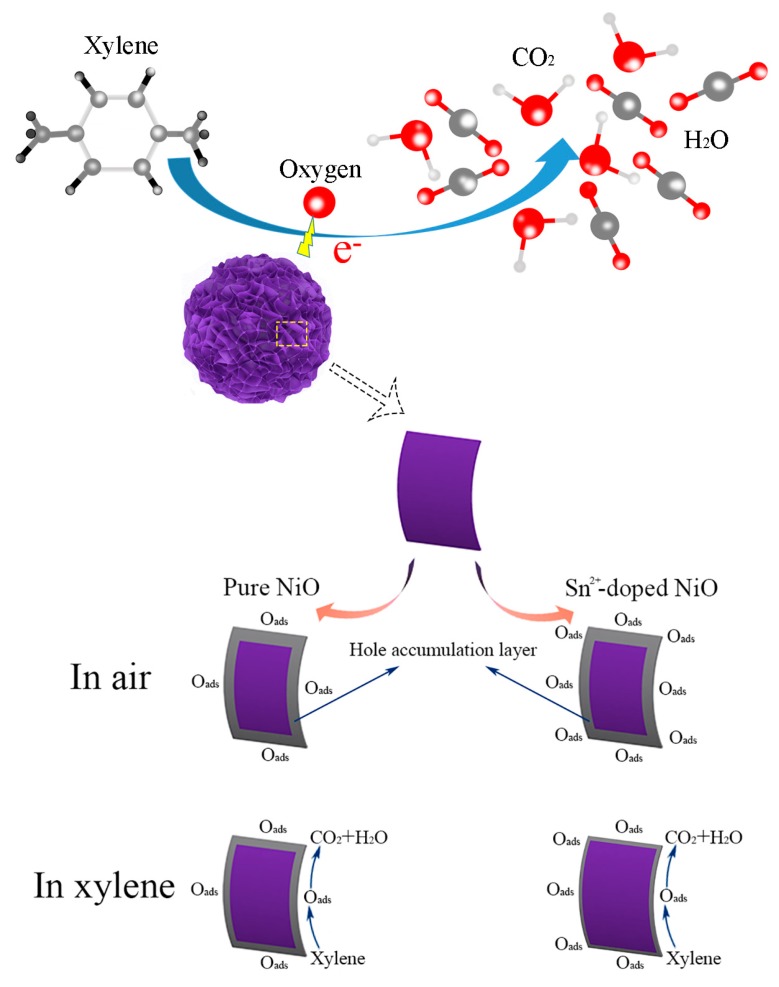
A schematic diagram of the proposed reaction mechanism of the SNM-8 sensor in air and in xylene, respectively.

As a typical p-type oxide, the main carriers in NiO and the SNM in these experiments are assumed to be positive holes. When NiO and the SNM are exposed to air, oxygen molecules adsorb on their surfaces, forming the chemisorbed oxygen species O_2_^−^ (T < 150 °C), O^−^ (150 C < T < 400 °C), and O^2−^ (T > 400 °C), which is accompanied by the capture of electrons from the conduction band and the consequent decrease in their resistance [33]. As illustrated in Figure 10, when NiO and the SNM are exposed to xylene, the adsorbed xylene is oxidized by the as-adsorbed oxygen species and transformed into CO_2_ and H_2_O. Simultaneously, the electrons extracted from xylene are transferred back to the conduction band, finally resulting in a decrease in the concentration of hole carriers and an increase in the resistance. The detailed reaction processes at a T_w_ of 180 °C can be expressed as follows [34]:

(2) $$O_{2}(\mathrm{gas})+e^{-}\to O_{2}^{-}(\mathrm{ads})$$

(3) $$O_{2}^{-}(\mathrm{ads})+e^{-}\to 2O^{-}(\mathrm{ads})$$

(4) $${\left(C_{6}H_{4}CH_{3}CH_{3}\right)}_{\mathrm{ads}}+2O^{-}\to (C_{6}H_{4}CH_{3}CHO)+H_{2}O(\mathrm{gas})+2e^{-}$$

(5) $$(C_{6}H_{4}CH_{3}CHO)+19O^{-}\to 8CO_{2}(\mathrm{gas})+4H_{2}O(\mathrm{gas})+19e^{-}$$

The reason for the improvement in the SNM performance is complex, but it is mainly due to the gain and loss of electrons and holes and their transport during gas adsorption, reaction and desorption. First, the change in the carrier concentration of the SNM might be a dominant factor, because it is typically two-times the order of magnitude of the change in the mobility due to Sn^2+^ doping [18]. Therefore, neglecting the change in the mobility, the gas response can be approximated as follows:

(6) $$S=\frac{R_{g}}{R_{a}}\approx \frac{P_{a}}{P_{g}}=\frac{P_{i}+j}{P_{i}-k}=1+\frac{j+k}{P_{i}-k}$$

where *P_g_*, *P_a_*, and *P_i_* are the hole concentrations of NiO (or the SNM) in the target gas, in air, and in a vacuum, respectively; *j* is the increase in the hole concentration due to the adsorbed oxygen in Equations (2) and (3); and *k* is the decrease in the hole concentration due to reducing gas adsorption (partially shown in Equations (4) and (5)). Therefore, the *j* and *k* values are mainly related to the surface adsorption properties, such as the oxidizing ability of the chemisorbed oxygen species. When Sn^2+^ is doped into the crystal lattice of NiO, the electronic compensation mechanism of the defect formation reaction due to interstitial doping can be expressed as follows [35]:

(7) $$\mathrm{SnO}\overset{\mathrm{NiO}}{\to }Sn_{i}^{••}+O_{o}^{\times }+2e^{\prime }$$

It can be seen that some electrons are generated to compensate for the positive charges of the interstitial Sn^2+^, which results in a decrease in the intrinsic hole concentration in the SNM relative to that in NiO. Furthermore, based on the contents of O_*II*_ and O_*III*_ in the XPS spectra, the SNM-x (x = 2, 4, and 8) samples can adsorb more oxygen to form the oxygen species and other gases for further reaction, i.e., the *j* and *k* values are higher for SNM-x than for NiO. Therefore, according to Equation (6), the decrease in P_i_ and the increases in the *j* and *k* values all positively contribute to the response. However, when the molar ratio of Sn^2+^/Ni^2+^ increases to 12.0%, the gas response starts to decrease. In fact, as discussed in the XPS section, Figure 5i shows that the contents of O_*II*_ and O_*III*_ in SNM-12 are lower than those in SNM-8. Accordingly, the *j* and *k* values of SNM-12 are also lower. Therefore, even if the P_i_ value of SNM-12 continues to decrease, the response might be reduced due to the decreases in the *j* and *k* values, according to Equation (6). Thus, it is possible for the response of SNM-12 to be smaller than that of SNM-8. Here, the decrease in the number of active sites and oxidizing ability of the adsorbed oxygen on SNM-12 might prevent the continued increase in the response. Furthermore, the relatively compact structure might also contribute to the degradation of the gas sensing performance. Compared to SNM-8, SNM-12 is denser, which might lead to a decrease in the gas accessibility [36]. Based on the XRD and TEM data, the average crystallite sizes of the SNM are smaller than that of the pure NiO. According to the literature [37], smaller particle sizes can enhance the sensitivity of a sensing material. However, in certain cases, this positive effect on the response might be counteracted by other factors, such as the surface properties discussed above. Generally, the overall mechanism of gas sensing is attributed to the competition of multiple factors.

## 4. Conclusions

In summary, SNM with different Sn^2+^ amounts were synthesized via a simple one-step hydrothermal method, and their gas sensing properties were investigated in detail. The results indicate that the SNM exhibit a significantly enhanced sensing performance toward xylene, and of all the SNM, SNM-8 has the highest response to 30 to 10 ppm xylene at 180 °C. This response is 21 times higher than that of the pure NiO. The detection limit is at the ppb level. The SNM also have a considerably improved selectivity for xylene detection. The significant improvement in the response to xylene can be attributed to the decrease in the intrinsic hole-carrier concentration, higher amounts of adsorbed oxygen and active sites, smaller crystallite sizes, and high gas accessibility with large specific surface areas. Considering the overall gas sensing characteristics such as the gas response, selectivity, and T_w_, the SNM-8 sensor with hierarchical nanostructures presented in this study provides a novel and promising solution for detecting xylene at the ppb level in practical applications in the future.

## Figures and Tables

**Table 1:** Gas sensing performance between this work and previous works in literatures.

Materials	Concentration (ppm)	Response R_g_/R_a_	Working Temperature (°C)	Tres/Trec (s)	Reference
NiO microspheres modified by Sn^2+^	10	3	250	500/1000 s	[17]
Sn-doped NiO microspheres	1	65.4	300	280/5000 s	[15]
Ni-doped ZnO nanowires	5	42.4	400	50/200 s	[5]
Cr-doped NiO nanostructures	10	5	325	144/50 s	[16]
W-doped NiO nanotubes	200	8.7	375	178/152 s	[11]
Pd-doped WO_3_·H_2_O nanomaterials	10	21	230	-	[7]
Cr-doped NiO nanostructures	5	24.5	425	-	[8]
α-MoO_3_ arrays	100	19.2	370	1/20 s	[28]
Co_3_O_4_ Nanofibers	100	10	255	15/22 s	[29]
Cr-loaded NiO micropheres	5	20.9	220	-	[30]
Co-ZnO nanofibers	100	14.8	320	4/6 s	[31]
Sn-doped NiO nanostructure	100	20.2	225	298/223 s	[32]
Sn^2+^-doped NiO micropheres	10	30	180	470/440 s	Our work
	900 ppb	1.6	180	700/200 s	
